# A student affairs podcast as novel communication tool

**Published:** 2019-11-28

**Authors:** Neda Frayha, Jessica Brown, Donna Parker

**Affiliations:** 1 University of Maryland School of Medicine, Maryland, USA

## Implication Statement

Podcasts are prevalent within medical education, but not within medical student affairs. Our Office of Student Affairs (OSA) created a podcast focusing on topics relevant to the medical student experience. There have been over 20,000 downloads thus far. Survey responses and feedback have been positive and highlight the podcast’s utility as a communication tool, with 96% of respondents saying they would recommend this podcast to others. Given the mission of student affairs offices to advise, mentor, and educate students, a student affairs podcast is an exciting innovation for medical schools to consider.

___

## Déclaration des répercussions

Les baladodiffusions prévalent en éducation médicale, mais pas auprès des organisations soutenant les affaires étudiantes. Notre Bureau des affaires étudiantes (BAE) a créé une baladodiffusion axée sur des sujets pertinents à l’expérience des étudiants en médecine. Il y a eu plus de 20 000 téléchargements jusqu’à maintenant. Les réponses au sondage et la rétroaction ont été positives et soulignent l’utilité des baladodiffusions comme outil de communication, avec 96 % des répondants disant qu’ils recommanderaient cette baladodiffusion aux autres. Étant donné que la mission des bureaux des affaires étudiantes est de conseiller, d’encadrer et d’éduquer les étudiants, une baladodiffusion des affaires étudiantes représente une innovation passionnante à envisager pour les facultésde médecine.

## Introduction

Podcasts are increasingly common in medical education for the delivery of specialty-specific content.^1^^-^^3^ However, podcasts from a Student Affairs perspective have been absent from the landscape.^1^^-^^3^ More than previous generations, Millennials expect teaching that is convenient and relevant in its delivery, and they listen to podcasts at higher rates than the general population.^4^^-^^6^ Given the mission of student affairs offices, a podcast is an innovative opportunity to connect and communicate with students beyond traditional methods such as lectures or email.

### Innovation

In February 2017, our Office of Student Affairs (OSA) created a podcast called *The OSA Insider*. The goals were to disseminate information relevant to student life and to improve the perception of the OSA as an approachable space.

The podcast’s launch required finding recording space and equipment; determining appropriate episode length and frequency; and selecting a media host to publish the podcast to popular platforms. An OSA assistant dean who possessed basic audio production skills served as producer and host.

Research regarding *The OSA Insider* and associated surveys was deemed exempt by the university’s Institutional Review Board in December 2017.

Students (n=16) participated in focus groups and completed surveys (n=172/648) about their podcast listening habits and preferences before the podcast’s launch. This feedback informed episode length, frequency, and topics. Ten months later, OSA sent out a survey about student reactions to the podcast and comfort levels in approaching the OSA with questions or concerns (n=106/644).

### Outcomes

As of July 2019, we have produced 44 unique podcast episodes of *The OSA Insider*, each lasting approximately 20 minutes and featuring interviews on topics including transitions in medical education, career choices, burnout, and wellness. Listeners have downloaded episodes over 20,000 times and have given enthusiastically positive feedback.

**Figure 1:**
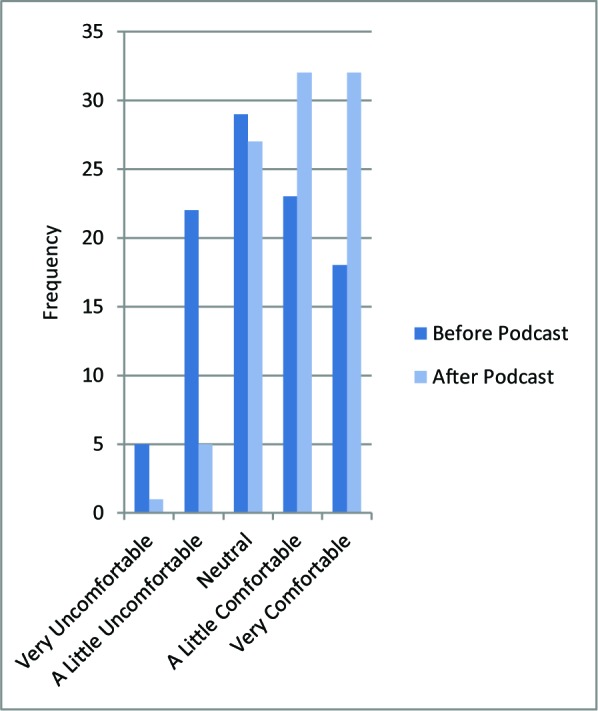
Student comfort in approaching the OSA with questions or concerns, pre- and post-podcast (n=97 responses)

Most survey respondents selected communication about important milestones and feeling less alone in their experiences as the best features of the podcast. Two survey questions asked respondents about comfort levels coming to the OSA before (M=3.3, SD=1.25) and then after (M=4.1, SD=0.95) listening to *The OSA Insider*. [Figure 1: Student Comfort] Students were significantly more comfortable coming to the OSA after the launch (paired t (82)=8.1, p <0.01, 95% CI: 0.56-0.93) when we excluded respondents who were neutral both before and after the podcast launch (13 out of 97 responses).

When asked if they would recommend this podcast, 96% (92/96) of students responded “yes.” OSA finds podcasting to be a convenient way to reach large numbers of students using a platform they enjoy.

### Next steps

This innovation's strengths include its novelty in the Student Affairs terrain, ease of accessibility, ability to address student questions as they evolve, fluidity of integration into the mission of Student Affairs offices, and subjective evaluations of its quality as an outreach tool. It requires relatively limited financial resources and faculty time; equipment such as a microphone and recorder can be purchased for approximately $500, and audio software programs are available at no cost. Average faculty time per episode is five hours. Potential limitations are that this is the experience of just one school, we performed a single follow-up survey, and most podcasting platforms do not provide data on who downloads episodes. Areas for future research include investigation into downloads by students versus other listeners, analysis of the most downloaded topics, surveying listenership on a regular basis, and collaborations with other medical schools.
